# Transcriptome analysis reveals the effect of oral contraceptive use on cervical cancer

**DOI:** 10.3892/mmr.2014.2466

**Published:** 2014-08-08

**Authors:** TIAN GAO, JIANJUN WANG, MIN YANG, HUAIFANG LI

**Affiliations:** 1 Department of Obstetrics and Gynecology, Tongji Hospital, Tongji University, Shanghai 200065, P.R. China; 2 Department of Obstetrics and Gynecology, Women’s Hospital School of Medicine, Zhejiang University, Hangzhou 310006, P.R. China

**Keywords:** cervical cancer, oral contraceptive, RNA-seq data, differentially expressed genes, biomarker

## Abstract

Differentially-expressed genes (DEGs) correlated to oral contraceptives (OCs) were identified by comparing the transcriptomes of cervical cancer patients who have taken OCs and those who have not. Their biological functions and relevance to clinical manifestations were investigated further in order to gain an understanding of the pathogenesis of cervical cancer and provide potential therapeutic targets. Level 3 RNA-sequencing (seq) data for cervical squamous-cell carcinoma and endocervical adenocarcinoma and the clinical information were downloaded from The Cancer Genome Atlas. The present study analyzed the RNA-seq data and information on OC use of 35 patients [OC users (n=18) and those who have never used OCs (n=17)]. Student’s t-test was used in order to identify DEGs and the false discovery rate (FDR) was estimated by a Beta-Uniform Mixture model, which was adopted in multiple testing corrections. A functional enrichment analysis was performed with the Database for Annotation, Visualization and Integrated Discovery tool and BioCarta. A total of 80 DEGs were identified in OC users while FDR=0.3 was set as the cut-off value. The metabolic process and human telomerase RNA gene transcription were significantly upregulated in DEGs. Furthermore, secreted LY6/PLAUR domain containing 1 was identified to be correlated to the pathological response, while the synapse defective 1 Rho GTPase homolog 2 was found to be significantly associated with the histological grade and overall survival time. In conclusion, present study shed light on the effect of OC use on the oncogenesis of the cervix and may indicate novel approaches for a targeted therapy of cervical cancer.

## Introduction

Cervical cancer is the second most common type of cancer in females worldwide (1). In China, there is an increasing trend in the incidence and mortality of cervical cancer. Numerous risk factors have been linked to this disease, including sexually transmitted diseases, sexual debut time, menstruation and childbirth, smoking, immune deficiency and viral infection (2).

Oral contraceptives (OCs) are one of the widely used contraceptive methods for females. As the main components of OCs are hormones (estrogen and progesterone), OC use may alter the gene expression and thereby increase the incidence of cervical cancer (3). Analysis of data from a total of 28 studies including 12,531 females with cervical cancer indicated that the risk of cervical cancer decreases following termination of OC use (4). Another pooled study including 16,573 females with and 35,509 with no cervical cancer reported that the relative risk of cervical cancer was increased in current users of OCs and declined after their use was terminated (5).

The mechanism underlying the increased risk of cervical cancer in OC users remains elusive. However, the most prevalent mechanism is a correlation between OC and human papilloma virus (HPV). HPV infection is thought to be the main but not the sole cause of the majority of cases of cervical cancer. Steroid contraception has been postulated to be one mechanism by which HPV exerts its tumorigenic effect on cervical tissue. It is believed that steroids bind to specific DNA sequences within transcriptional regulatory regions on the HPV DNA, either to increase or suppress the transcription of various genes (6). In females using combined OCs, the cervical mucus remains scanty, thick and highly viscous (7). Therefore, Guven *et al* (8) hypothesized that cervical mucus changes may contribute to the pathogenesis of cervical cancer, since scanty, thick and highly viscous cervical mucus may modulate and prolong the effect of carcinogenic agents.

To assess changes in global gene expression resulting from OC use, the transcriptome of cervical cancer patients with and without OC use were compared and differentially-expressed genes (DEGs) correlated to OC use were identified. Further investigations were performed on their biological functions and their relevance to clinical manifestations, which may be beneficial for the development of novel therapies.

## Materials and methods

### RNA-seq data and clinical information

Level 3 RNA-seq data, which had been obtained using a Illumina HiSeq (Illumina, San Diego, CA, USA), for cervical squamous-cell carcinoma and endocervical adenocarcinoma as well as the clinical information were downloaded from The Cancer Genome Atlas (TCGA; National Human Genome Research Institute, National Institute of Health, Bethesda, MD, USA; http://cancergenome.nih.gov/). The raw data provided reads per kilobase of exon per million mapped reads (RPKM, a measure of relative gene expression) for each gene. Data for 35 samples were obtained (17 samples from patients who never used OC, 16 samples from former users and 2 samples from current users). Given the small sample size, samples from former and present users were combined. Clinical data were also collected, including the time of diagnosis, time of death and follow-up time, from which the survival time could be calculated. The pathological response status, histological grade, age and race were also contained in the Biotab file.

### Screening of DEGs

R software, version 3.0.1 (9) was used for statistical analysis. A t-test was used to identify DEGs. In multiple testing corrections, the false discovery rates (FDRs) were estimated using a Beta-Uniform Mixture (BUM) model (10).

### Functional enrichment analysis

Gene Ontology (GO) enrichment analysis was performed with the Database for Annotation, Visualization and Integrated Discovery tool (DAVID; National Institute of Health; http://david.abcc.ncifcrf.gov/) (11) with hypergeometric distribution. The P-value was adjusted by the Benjamini & Hochberg method (12). BioCarta (San Diego, CA, USA; http://www.biocarta.com) was also used to identify enriched regulatory networks in DEGs. All statistical analyses were conducted using R software, version 3.0.1 (9)

### Gene expression and clinical manifestations

The relevance of gene expression to clinical manifestations was investigated. The t-test was applied to examine the correlation between genes and the pathological response. Analysis of variance (ANOVA) was used to analyze the gene expression among different histological grades. The Cox model was selected to explore the significance of the correlation between gene expression and the survival time.

## Results

### Differentially-expressed genes

**Figure 1:**
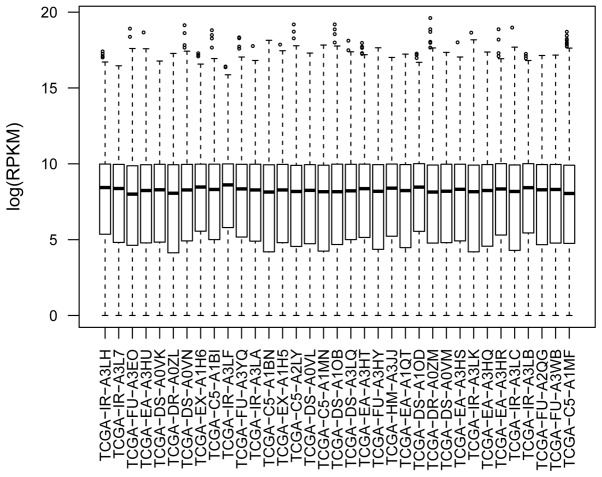
Box plot for the RNA-seq data. RPKM, reads per kilobase of exon per million mapped reads.

**Figure 2:**
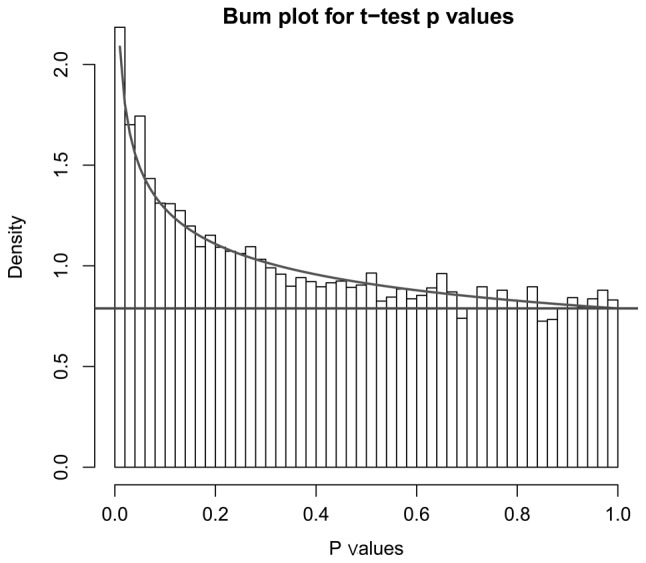
Distribution of P-values and fitted BUM curve. BUM, Beta-Uniform Mixture.

**Figure 3:**
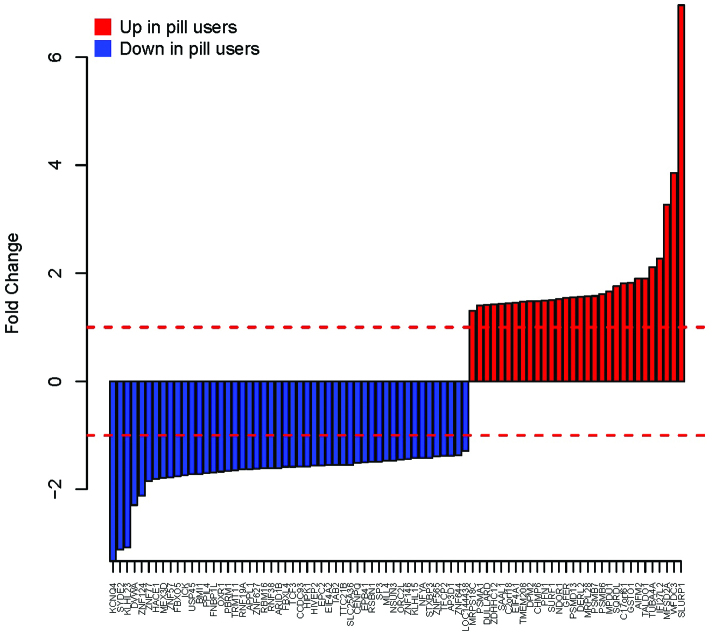
Fold changes for each differentially-expressed gene.

The distribution of RPKM was similar for each sample following data pre-treatment (Fig. 1). DEGs were screened using the t-test. The distribution of the P-value and the fitted BUM curve are shown in Fig. 2. A total of 80 DEGs were disclosed, while FDR=0.3 was set as the threshold value. The fold changes of these genes are shown in Fig. 3.

### Cluster analysis results

**Figure 4:**
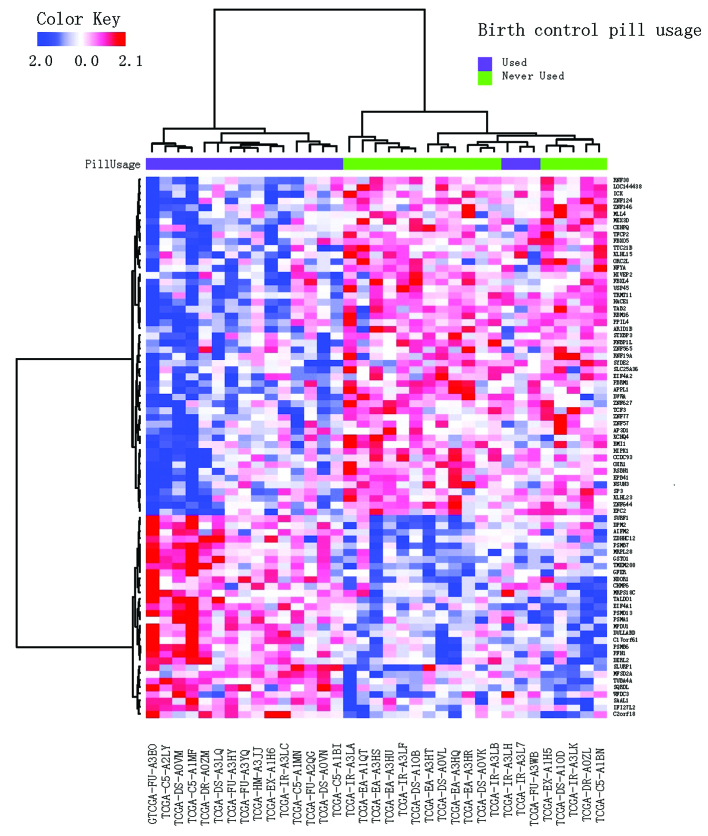
Cluster analysis result.

A cluster analysis was performed using DEGs. The distance was defined as 1-Pearson correlation coefficient and the Ward’s method was applied in hierarchical clustering. The result is shown in Fig. 4. A total of three samples from former OC users were grouped with the control. It is speculated that the disturbance of gene expression by OCs may diminish with time.

### Enriched biological functions

**Figure 5:**
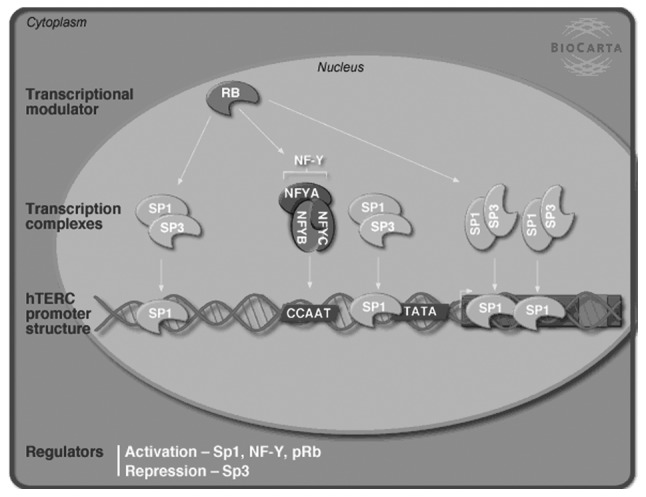
hTERC transcriptional regulation was enriched in differentially-expressed genes according to the BioCarta analysis. hTERC, human telomerase RNA gene; pRB, retinoblastoma protein; NF-Y, nuclear transcription factor Y; SP, specificity protein.

A GO enrichment analysis indicated that cellular macromolecule metabolic processes were over-represented in DEGs (Table I). BioCarta analysis indicated that DEGs were involved in the transcriptional regulation of the human telomerase RNA gene (hTERC) (Fig. 5; P=0.038, Fisher’s exact test).

### Relevance of secreted LY6/PLAUR domain containing 1 (SLURP1) and synapse defective 1 Rho GTPase homolog 2 (SYDE2) to clinical manifestations

**Figure 6:**
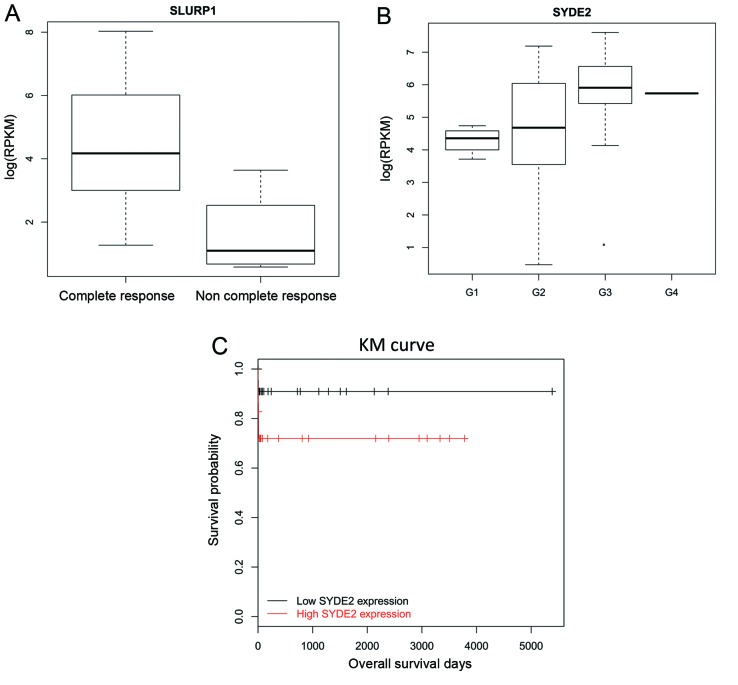
Relevance of SLURP1 and SYDE2 to clinical manifestations. (A) Relevance of SLURP1 to pathologic response (P=0.014, t-test); (B) relevance of SYDE2 to histological tumor grade (P=0.0028, analysis of variance) (G1–4, grades 1–4); (C) relevance of SYDE2 to survival time (P=0.042, Log Rank test). SLURP1, secreted LY6/PLAUR domain containing 1; SYDE2, synapse defective 1 Rho GTPase homolog 2; KM, Kaplan-Meier.

SLURP1 was the most upregulated gene, whereas SYDE2 was the second most downregulated gene. Their relevance to clinical manifestations was explored. SLURP1 was correlated to the pathological response (Fig. 6; P=0.014, t-test), while SYDE2 was associated with the histological grade (P=0.0028, ANOVA) and survival time (P=0.042, Log Rank test). An upregulation of SLURP1 was observed in OC users and it was linked to the reduced frequency of the complete response (Fig. 6A). Low expression of SYDE2 was observed in patients with histological grade 1 tumors (Fig. 6B) and it was linked with a longer survival time (Fig. 6C). Therefore, it was believed that SLURP1 and SYDE2 were potential therapeutic targets for cervical cancer. Additionally, the inhibition of SYDE2 may improve the survival time.

## Discussion

In the present study, the transcriptome of cervical cancer patients with and without OC use were compared in order to identify any relevant DEGs. A total of 80 genes were identified as DEGs, while FDR=0.3 was set as the cut-off value. This finding confirmed that OC use had a large impact on the gene expression in the cervix and thereby stimulated the pathogenesis of cervical cancer. GO enrichment analysis revealed that the metabolic process was enriched in the DEGs. BioCarta analysis indicated that DEGs were involved in the hTERC transcriptional regulation.

This finding was in accordance with previous studies, which have indicated that OCs have an impact on the lipid and carbohydrate metabolism (13). Hormones present in OCs significantly affect the plasma lipoprotein metabolism, which can raise the levels of plasma triglycerides, low-density lipoprotein, and high-density lipoprotein 3. They also affect the carbohydrate metabolism, primarily through the activity of progestin, causing conditions including insulin resistance, increases in plasma insulin levels and relative glucose intolerance (14).

Further interesting DEGs were identified, including adaptor-related protein complex 3 delta 1 (AP3D1). Downregulation of genes encoding for subunits of adaptor complex-3 has been reported in cervical cancer compared with normal controls (15). In the present study, AP3D1 was identified to be further downregulated in the OC group compared with non-OC group. AP3D1 is a subunit of the AP3 adaptor-like complex, which is associated with the golgi region and more peripheral structures. The AP-3 complex facilitates the budding of vesicles from the golgi membrane, and may be directly involved in trafficking to lysosomes (16). BMI1 polycomb ring finger oncogene (BMI1) is a member of the polycomb group, which participates in axial patterning, hematopoiesis, cell cycle regulation and senescence. It may contribute to malignant cell transformation and its overexpression has been reported in cervical cancer (17,18). However, according to the present study, BMI1 was downregulated in OC users. Further studies on these genes may be helpful in elucidating the molecular mechanism.

Telomerase is essential for the immortalization of the majority of human cancer cells. A study by Guilleret *et al* (19) indicated that hTERC expression is regulated during carcinogenesis. This gene is often amplified in certain types of solid human tumors, including the cervix (20). Guo *et al* (21) reported that hTERC amplification testing is a promising evaluation method for cervical cancer screening. This gene may be activated by the transcription complex nuclear transcription factor (NF)-Y as well as the transcription factors specificity protein (Sp)1 and retinoblastoma protein (pRB) and could be repressed by Sp3 (22). In the present study, Sp3 was downregulated in the OC group, which may contribute to the increased incidence of cervical cancer through pathways including hTERC. NF-Y alpha was also downregulated in OC users. Therefore, further studies are required in order to elucidate the effect of OC use on the hTERC transcriptional regulation.

SLURP1 and SYDE2 exhibited considerable fold changes in OC users and thereby, their relevance to clinical manifestations was investigated in the present study. The expression of SLURP1 was identified to be negatively correlated with the pathological response, whereas the expression of SYDE2 was negatively correlated with the survival time. SLURP1 and SYDE2 may be potential therapeutic targets for cervical cancer.

SLURP1 is a member of the Ly6/uPAR family that lacks a GPI-anchoring signal sequence. It is thought that this secreted protein contains antitumor activity. A study by Pettersson *et al* (23) indicated that SLURP1 participates in the regulation of the gut immune functions and motility, as well as possibly having a role in colon carcinogenesis/cancer progression. Kalantari-Dehaghi *et al* (24) also identified that cancer-associated genes upregulation by nitrosamine 4-(methylnitrosamino)-1-(3-pyridyl)-1-butanone can be abolished by SLURP1 (24). SYDE2, a GTPase activator, has also been reported to be a tumor suppressor (25). Methylated SYDE1 was demonstrated to be significantly associated with the relapse-free survival of patients with breast cancer (26) However, there are currently no studies regarding the implications of SLURP1 and SYDE2 in cervical cancer. Therefore, further studies should investigate their roles in this cervical cancer.

In conclusion, the present study identified a range of DEGs associated with OC use in patients with cervical cancer. Further study of these genes may aid in elucidating the underlying mechanism of the generation of cervical cancer associated with OC use. Furthermore, SLURP1 and SYDE2 may be potential therapeutic targets for cervical cancer.

## Figures and Tables

**Table I:** Gene Ontology (GO) enrichment analysis result for the differentially-expressed genes.

Category	Term	Count (n)	P-value	Adjusted P-value^a^
GOTERM_BP_FAT	Protein catabolic process	11	5.50×10^−4^	7.20×10^−2^
GOTERM_BP_FAT	Cellular protein catabolic process	11	4.30×10^−4^	7.50×10^−2^
GOTERM_CC_ALL	Intracellular	64	3.20×10^−5^	2.60×10^−3^
GOTERM_CC_ALL	Intracellular membrane-bounded organelle	53	3.20×10^−5^	1.80×10^−3^
GOTERM_CC_ALL	Membrane-bounded organelle	53	3.30×10^−5^	1.40×10^−3^
GOTERM_CC_ALL	Intracellular organelle	55	2.40×10^−5^	8.00×10^−3^
GOTERM_CC_ALL	Organelle	55	2.50×10^−5^	7.00×10^−3^
GOTERM_BP_ALL	Cellular macromolecule metabolic process	40	4.80×10^−5^	3.10×10^−2^
GOTERM_BP_ALL	Macromolecule metabolic process	41	1.80×10^−5^	5.70×10^−2^
GOTERM_BP_ALL	Metabolic process	49	2.50×10^−5^	5.30×10^−2^

a P-value was adjusted with the Benjamini & Hochberg method.

## References

[1] Brake T, Lambert PF (2005). Estrogen contributes to the onset, persistence, and malignant progression of cervical cancer in a human papillomavirus-transgenic mouse model. Proc Natl Acad Sci USA.

[2] Kjellberg L, Hallmans G, Hren A (2000). Smoking, diet, pregnancy and oral contraceptive use as risk factors for cervical intra-epithelial neoplasia in relation to human papillomavirus infection. Br J Cancer.

[3] Ness RB, Grisso JA, Klapper J (2000). Risk of ovarian cancer in relation to estrogen and progestin dose and use characteristics of oral contraceptives. Am J Epidemiol.

[4] Smith JS, Green J, De Gonzalez AB (2003). Cervical cancer and use of hormonal contraceptives: a systematic review. The Lancet.

[5] Sasieni P (2007). Cervical cancer and hormonal contraceptives: Collaborative reanalysis of individual data for 16 573 women with cervical cancer and 35 509 women without cervical cancer from 24 epidemiological studies. Commentary Lancet.

[6] Moodley M, Moodley J, Chetty R, Herrington C (2003). The role of steroid contraceptive hormones in the pathogenesis of invasive cervical cancer: a review. Int J Gynecol Cancer.

[7] Rivera R, Yacobson I, Grimes D (1999). The mechanism of action of hormonal contraceptives and intrauterine contraceptive devices. Am J Obstet Gynecol.

[8] Guven S, Kart C, Guvendag Guven ES, Serdar Gunalp G (2007). The underlying cause of cervical cancer in oral contraceptive users may be related to cervical mucus changes. Med Hypotheses.

[9] The R Development Core Team R: A language and environment for statistical computing.

[10] Pounds S, Morris SW (2003). Estimating the occurrence of false positives and false negatives in microarray studies by approximating and partitioning the empirical distribution of p-values. Bioinformatics.

[11] Da Wei Huang BTS, Lempicki RA (2008). Systematic and integrative analysis of large gene lists using DAVID bioinformatics resources. Nat Protoc.

[12] Benjamini Y, Hochberg Y (1995). Controlling the false discovery rate: a practical and powerful approach to multiple testing. J R Stat Soc Series B Stat Methodol.

[13] Godsland IF, Crook D, Simpson R (1990). The effects of different formulations of oral contraceptive agents on lipid and carbohydrate metabolism. N Engl J Med.

[14] Krauss RM, Burkman R (1992). The metabolic impact of oral contraceptives. Am J Obstet Gynecol.

[15] Petrenko A, Pavlova L, Karseladze A, Kisseljov F, Kisseljova N (2006). Downregulation of genes encoding for subunits of adaptor complex-3 in cervical carcinomas. Biochemistry (Moscow).

[16] Odorizzi G, Cowles CR, Emr SD (1998). The AP-3 complex: a coat of many colours. Trends Cell Biol.

[17] Honig A, Weidler C, Häusler S (2010). Overexpression of polycomb protein BMI-1 in human specimens of breast, ovarian, endometrial and cervical cancer. Anticancer Res.

[18] Gavrilescu MM, Todosi AM, Anitei MG, Filip B, Scripcariu V (2012). Expression of bmi-1 protein in cervical, breast and ovarian cancer. Rev Med Chir Soc Med Nat Iasi.

[19] Guilleret I, Yan P, Guillou L, Braunschweig R, Coindre J-M, Benhattar J (2002). The human telomerase RNA gene (hTERC) is regulated during carcinogenesis but is not dependent on DNA methylation. Carcinogenesis.

[20] Cairney C, Keith W (2008). Telomerase redefined: integrated regulation of hTR and hTERT for telomere maintenance and telomerase activity. Biochimie.

[21] Guo Q, Sui L, Feng Y (2012). Cervical cancer screening: hTERC gene amplification detection by FISH in comparison with conventional methods. Open Journal of Obstetrics and Gynecology.

[22] Zhao JQ, Glasspool RM, Hoare SF (2000). Activation of telomerase rna gene promoter activity by NF-Y, Sp1, and the retinoblastoma protein and repression by Sp3. Neoplasia.

[23] Pettersson A, Nordlander S, Nylund G (2008). Expression of the endogenous, nicotinic acetylcholine receptor ligand, SLURP-1, in human colon cancer. Auton Autacoid Pharmacol.

[24] Kalantari-Dehaghi M, Bernard HU, Grando SA (2012). Reciprocal effects of NNK and SLURP-1 on oncogene expression in target epithelial cells. Life Sci.

[25] Iorns E, Ward TM, Dean S (2012). Whole genome in vivo RNAi screening identifies the leukemia inhibitory factor receptor as a novel breast tumor suppressor. Breast Cancer Res Treat.

[26] Hill VK, Ricketts C, Bieche I (2011). Genome-wide DNA methylation profiling of CpG islands in breast cancer identifies novel genes associated with tumorigenicity. Cancer Res.

